# Comparative metabolomic analysis reveals shared and unique chemical interactions in sponge holobionts

**DOI:** 10.1186/s40168-021-01220-9

**Published:** 2022-02-01

**Authors:** Shan Zhang, Weizhi Song, Louis-Félix Nothias, Sneha P. Couvillion, Nicole Webster, Torsten Thomas

**Affiliations:** 1 School of Biotechnology and Biomolecular Sciences, University of New South Wales, Sydney, 2052 Australia; 2 Centre for Marine Science and Innovation, University of New South Wales, Sydney, 2052 Australia; 3 School of Biological, Earth and Environmental Sciences, University of New South Wales, Sydney, 2052 Australia; 4 School of Pharmacy and Pharmaceutical Sciences, University of California San Diego, La Jolla, CA USA; 5 Biological Sciences Division, Pacific Northwest National Laboratory, Richland, WA USA; 6 Australian Institute of Marine Science, Townsville, Australia; 7 Australian Centre for Ecogenomics, The University of Queensland, Brisbane, Australia

**Keywords:** Sponge, Metabolomics, Symbionts, Antioxidants, Antagonist

## Abstract

**Background:**

Sponges are ancient sessile metazoans, which form with their associated microbial symbionts a complex functional unit called a holobiont. Sponges are a rich source of chemical diversity; however, there is limited knowledge of which holobiont members produce certain metabolites and how they may contribute to chemical interactions. To address this issue, we applied non-targeted liquid chromatography tandem mass spectrometry (LC-MS/MS) and gas chromatography mass spectrometry (GC-MS) to either whole sponge tissue or fractionated microbial cells from six different, co-occurring sponge species.

**Results:**

Several metabolites were commonly found or enriched in whole sponge tissue, supporting the notion that sponge cells produce them. These include 2-methylbutyryl-carnitine, hexanoyl-carnitine and various carbohydrates, which may be potential food sources for microorganisms, as well as the antagonistic compounds hymenialdisine and eicosatrienoic acid methyl ester. Metabolites that were mostly observed or enriched in microbial cells include the antioxidant didodecyl 3,3′-thiodipropionate, the antagonistic compounds docosatetraenoic acid, and immune-suppressor phenylethylamide. This suggests that these compounds are mainly produced by the microbial members in the sponge holobiont, and are potentially either involved in inter-microbial competitions or in defenses against intruding organisms.

**Conclusions:**

This study shows how different chemical functionality is compartmentalized between sponge hosts and their microbial symbionts and provides new insights into how chemical interactions underpin the function of sponge holobionts.

Video abstract

**Supplementary Information:**

The online version contains supplementary material available at 10.1186/s40168-021-01220-9.

## Background

Sponges (phylum Porifera) are multicellular animals that have been evolving since the Precambrian [1]. Sponges often form stable associations with diverse, abundant and species-specific microbial communities, which can account for up to 35% of the total biomass of the sponge [2–4]. Mutualistic or commensal interactions contribute to the complex functional unit comprising the sponges and its associated microbial symbionts, which is often referred to as a metaorganism or holobiont [4]. Specifically, studies have found that sponge-associated microbial symbionts play key roles in maintaining the health of their hosts by providing organic nutrients [5–9], eliminating toxic metabolic by-products [10], protecting the hosts against oxidative stress [11], inhibiting parasites and pathogens [12], and preventing cellular damage by screening out UV radiation [13]. Symbionts may in return benefit by obtaining nutrients [14, 15] and shelter through their hosts [16].

Sponges are a rich source of natural products, and traditional analytical techniques as well as bioassay-guided fractionation have been extensively used to uncover a remarkable metabolite diversity, including unusual nucleosides, terpenes, sterols, cyclic peptides, alkaloids, fatty acids, peroxides, and amino acid derivatives ([17–20] and MarinLit database http://pubs.rsc.org/marinlit/). A wide range of therapeutic properties have been ascribed to these natural products, including as enzyme regulators and inhibitors [21, 22] or as antimicrobial [23–26], anti-inflammatory [27], anticancer [24, 28], antitumor [29], anti-atherosclerotic [30], or antiherpetic [31] agents.

Some metabolites also possess important direct biological and ecological benefits for the sponge. For example, lysophospholipids (LPLs) may act as signaling molecules in the embryogenesis and morphogenesis of the sponge *Oscarella tuberculate* by regulating cell division and differentiation, or by simply being a lipid reserve [32]. Chemical defense molecules have also been extensively studied in sponges. For instance, latrunculin-A, a toxic compound from the sponge *Latrunculia magnifica*, can repel and kill fish [33]. Additionally, the siphonodictidin-containing mucus secreted by the sponge *Siphonodictyon coralliphagum* has been found to inhibit coral growth [34], while idiadione and heteronemin that are exuded by the sponge *Leiosella idia* can prevent fouling organisms from settling or overgrowing the sponge [35]. However, the biological and ecological functions of most sponge-derived metabolites are poorly understood in comparison to their therapeutic or pharmacological applications.

Most chemical analyses have utilized extracts of whole sponge tissue (i.e., the mixture of sponge cells and microbial cells) and the sponge cells themselves have often been intuitively regarded as the producers of any metabolite found [36–39]. However, the observation that a number of sponge-derived metabolites are structurally similar to those found in microorganisms has shifted the focus to explore microbial symbionts as major contributors to the chemical diversity found in sponges [40–45]. For example, the structure of sponge-derived halichondrin B has been found to resemble the structure of dinoflagellate-derived polyketides [41]. The presence of genes for the biosynthetic pathways or key enzyme for the synthesis of sarasinoside [46], polybrominated diphenyl ethers [47], and butenolides [26] in sponge-associated microbial symbionts also has further supported this notion. However, further work is required to define the true producers of metabolites to understand how specific members contribute to chemical functionalities in sponge holobionts.

Non-targeted metabolomics involving mass spectrometry (MS) and tandem MS in combination with liquid chromatography (LC) or gas chromatography (GC) are powerful approaches to profile the metabolite content of complex biological samples [48, 49]. Recent studies have applied non-targeted metabolomics to probe intraspecific, interspecific, and environmental variation of metabolite profiles in sponges. For example, the metabolomes of various species of *Xestospongia* were found to differ across four geographical locations and several metabolites were associated with specific environmental conditions [50]. In another study, seasonal changes as well as responses to increased water temperature were observed in the metabolomes of *Haliclona fulva* and *H. mucosa* [51]. Furthermore, comparative metabolomic analysis of two geographically co-located sponges (*Melophlus sarasinorum* and *Ianthella basta*) revealed very little metabolomic overlap, which was speculated to be due to the substantial differences in their associated microbial symbionts [46]. Further studies are however required to understand the variability and similarity between as well as within various sponge species.

Here, we applied non-targeted LC-MS/MS and GC-MS to define common and unique metabolites in six sponge species (Carteriospongia foliascens, Cliona orientalis, Coscinoderma matthewsi, Ircinia ramosa, Pericharax heteroaphis, and Stylissa flabelliformis) that co-occur on the Great Barrier Reef, Australia, to further the knowledge on metabolome variability in sponges. Metabolite profiles from isolated microbial cells and whole sponge tissue were analyzed to determine the likely producers of these metabolites within the holobiont. To provide an improved understanding of the diverse suite of chemical interactions taking place in sponge holobionts, our observations are interpreted in the context of known metabolite functions.

## Methods

### Sample collection and processing

The marine sponges *Carteriospongia foliascens*, *Cliona orientalis*, *Coscinoderma matthewsi*, *Ircinia ramosa*, *Pericharax heteroaphis*, and *Stylissa flabelliformis* were collected at Davies Reef on the Great Barrier Reef, Queensland, Australia (latitude −18.8225, longitude 147.6375) on the 22nd and 23rd of December 2015 using previously established methods [52]. Four biological replicates of each sponge species were collected on SCUBA at a depth of 5‑9 m and brought back to the surface in separated plastic bags filled with seawater. Part of the tissue from each specimen was immediately preserved in 70% ethanol for species classification and the remaining tissue was snap-frozen in liquid nitrogen. Sponge species were identified based on their morphological characters [53] by taxonomic specialists at the Western Australian Museum (Perth, Australia).

The frozen tissue samples were rinsed with sterile seawater and cut into small cubes (~1 cm^3^). Half of the cubes were used for whole sponge tissue analysis, while the rest were used to physically fractionate microbial cells from sponge cells using established procedures [52]. Briefly, the sponge cubes were homogenized, then filtered through a 100-μm nylon sterile cell strainer (Corning, New York, USA) and the flowthrough was centrifuged to obtain the supernatant, which was then successively filtered through 8 μm and 5 μm membrane filters. Recovered microbial cells were then pelleted, rinsed twice in sterile buffer (10 mM Tris HCl at pH 8 and 0.5 M NaCl), pelleted again, and finally resuspended in 1 mL of sterile buffer. Cells were stored at −20 °C.

### Sample preparation and LC-MS/MS analysis

Samples were prepared and extracted following the Earth Microbiome Project (EMP) protocol [54]. Briefly, whole sponge tissue samples and microbial cell pellets were resuspended in 7:3 MeOH:H_2_O, homogenized in a tissue-lyser (QIAGEN) and centrifuged. The supernatant was collected, and salts and the most apolar compounds were removed by solid phase extraction (SPE) with a mixed hydrophilic-lipophilic stationary phase. Samples were analyzed by reversed-phase liquid chromatography (UHPLC) (Vanquish, Thermo Scientific) coupled to a quadrupole-Orbitrap mass spectrometer (Q Exactive, Thermo Scientific) operating in data-dependent acquisition (DDA) mode.

The LC-MS/MS spectrometry data were processed and annotated following the EMP method [55]. Briefly, the proprietary files were converted to the mzML open format, processed with GNPS classical molecular networking workflow [56, 57]. The results can be accessed at https://gnps.ucsd.edu/ProteoSAFe/status.jsp?task=eda585e3157645179395acfdfd962055 for the original job, and https://gnps.ucsd.edu/ProteoSAFe/status.jsp?task=31a1dda1224347b7beec3842d17b873e for the most recent version of the workflow that offer Metabolomics USI views [58]. The mass spectrometry data were deposited on the MassIVE public repository under the accession MSV000083475. Spectral library matching was performed against public MS/MS spectral libraries available on GNPS and the NIST17 library to obtain level 2 annotations (putative structure or related (stereo)isomer) based on the metabolomics standard initiative (MSI) standards [59]. The mass spectra and the spectral matching results are accessible with the Metabolomics USI interface using the GNPS job ID and the corresponding MF id (e.g., X4151 corresponds to the scan/cluster number 4151). The computational annotations of putative small peptides, which can be classified as level 2/3 annotation (putative/partial structure) based on MSI standards [59], were performed using the DEREPLICATOR algorithm v.1.2.8 [60] on GNPS. Results of the DEREPLICATOR workflow can be accessed at https://gnps.ucsd.edu/ProteoSAFe/status.jsp?task=31be4f1f9d2e46a99823811ddd0cfd70.

### Sample preparation and GC-MS analysis

Metabolites were extracted from the samples using the MPLEx protocol, a modified Folch extraction method, as described previously [61]. A detailed description of the sample preparation can be found at [62]. Briefly, whole sponge tissue samples and microbial cell pellets were resuspended in 3:8:4 H_2_O:CHCl_3_:MeOH, then homogenized, and centrifuged. The middle layer was collected, dried in a vacuum concentrator, and chemically derivatized using a modified version of the protocol described by Kind et al. [63]. Specifically, the derivatization process was performed by evaporating extract to dryness. Methoxyamine solution was then added and incubated at 37 °C for 2 h. Samples were then injected in splitless mode into an Agilent 7890A gas chromatograph coupled fitted with a single quadrupole 5975C mass spectrometer (Agilent Technologies, Inc.).

The GC-MS data were processed following the EMP method [55]. Briefly, the proprietary files were converted to netCDF file format and the results files were processed with MetaboliteDetector [64] to detect, align, and measure metabolite intensities across samples. The mass spectrometry data were deposited on the MassIVE public repository under the accession number MSV000083743. Metabolites were identified by matching measured retention indices (RI) and mass spectra to an augmented version of the Agilent Fiehn Metabolomics Retention Time Locked (RTL) Library [63]. The NIST 08 GC-MS library was also used to cross-validate the spectral matching scores obtained using the Agilent library. The metabolite intensities and identification results can be accessed at ftp://massive.ucsd.edu/MSV000083743/updates/2019-08-22_lfnothias_7cc043bc/other/. The QC files for each samples can be inspected at ftp://massive.ucsd.edu/MSV000083743/other/.

### Blank subtraction, data filtering, and normalization

Molecular features (MFs) of the LC-MS/MS dataset with peak ion intensity two folds higher than their mean value across ten blank samples were kept. MFs of the GC-MS dataset were retained unless their peak ion intensity was higher than that in any of nine blank samples. MFs that were present in only one sample were removed from further analysis. All missing variables of ion intensity were replaced by the limit of detection (LoDs; 1/5 of the minimum positive value) using MetaboAnalyst v.3.0 [65]. Ion intensity per MF was normalized by dividing it by the sum of all MFs’ intensity values in a sample.

### Statistical analysis and data visualization

Permutational multivariate analysis of variance (PERMANOVA, R: vegan v2.5-6, 999 permutations) using Euclidean distances of normalized MFs data was used to statistically evaluate overall differences in metabolite profiles, and the cluster dendrogram and principal component analysis (PCA, R: prcomp()) was used to visualize the data. Cluster dendrogram was plotted using Ward’s minimum variance method (ward.D2) [66]. Common and unique MFs across the six sponge species were visualized using Venn diagrams (R: venn v1.9).

Normality and homoscedasticity of MFs were assessed by Shapiro-Wilk’s test and Levene’s test, respectively [67]. The percentage of MFs that rejected H_0_ at conventional *α* = 0.05 relative to the total number of MFs is presented in Supplemental Table S1. Differential abundance analysis of MFs between sample types was performed by applying either the unpaired *t* test (parametric, normal distribution) or Mann-Whitney test (non-parametric, non-normal distribution) to the MFs, followed by multiple testing correction (Benjamini-Hochberg Procedure). Heatmaps of annotated differentially abundant MFs (aDAMs) were plotted with the cube-root relative abundance of the ion intensity (R: pheatmap v1.0.1).

## Results and discussion

### Overview of the dataset

The non-targeted LC-MS/MS metabolomic dataset contained a total of 10,118 MFs from the whole sponge tissue (ST) and microbial cell (MC) samples across the six sponge species, and 3422 of them were detected in at least two samples and were used for data normalization and downstream analysis (Table 1). The ST samples contained overall more MFs (2758) compared to the MC samples (2129), which is not surprising given that metabolites found in the microbial cells would also be part of the whole sponge tissue. However, only 53% and 69% of the MFs in the ST and MC samples overlapped between the two sample types, respectively. The metabolite diversity per species ranged from 773 for *C. matthewsi* to 1009 MFs for *P. heteroaphis*.

**Table 1:** Overall number of MFs in different sample types for each sponge species. MFs were included after application of blank subtraction and data filtering. Differentially abundant molecular features (DAMs) detected from the LC-MS/MS analysis were obtained from subsets belonging to each sponge species, while those detected from the CG-MS analysis were obtained across all species (marked with an asterisk) (see below in the “Shared or differential abundant metabolites” section)

Sponge species		All species combined		***C. foliascens***		***C. orientalis***		***C. matthewsi***		***I. ramosa***		***P. heteroaphis***		***S. flabelliformis***	
Sample types		ST	MC	ST	MC	ST	MC	ST	MC	ST	MC	ST	MC	ST	MC
**LC-MS/MS**	**# Samples in each sample type**	24	20	4	3	4	4	4	3	4	3	4	4	4	3
	**# MFs found**	3422		870		907		773		889		1009		858	
	**# DAMs between ST and MC**	N/A		206		139		119		8		43		303	
	**# aDAMs between ST and MC**	N/A		13		29		17		2		7		36	
	**# aDAMs more abundant in the sample type**	N/A		2	11	1	28	15	2	0	2	0	7	1	35
**GC-MS**	**# Samples in each sample type**	12	11	2	2	2	2	2	2	2	1	2	2	2	2
	**# MFs found**	503		357		373		373		358		379		346	
	# DAMs between ST and MC^*^	60		N/A											
	# aDAMs between ST and MC^*^	15		N/A											

*aDAMs* Annotated DAMs, *N/A* Not applicable

Of the 3422 MFs, 229 could be assigned to level 2 annotations with scores between 0.6 and 1. Thirteen MFs were assigned to level 2/3 annotations with *p* values between 2.2E−34 and 6.9E−12. These combined 242 annotated MFs (aMFs) were subjected to biological interpretations in the differential abundance analysis (see below in section the “Shared or differential abundant metabolites” section and Additional file 6: Appendix file 1 for more details).

The non-targeted GC-MS analysis yielded a total of 538 MFs from ST and MC samples across the six sponge species, and 503 of those were detected in at least two samples and were therefore normalized and further analyzed (Table 1). Similar to the LC-MS/MS analysis, more MFs were detected from the ST samples (477) in comparison to the MC samples (442). However, a much higher proportion of MFs was found to overlap between the ST and MC samples (87% and 94%, respectively) in comparison to the LC-MS/MS analysis. The metabolite diversity per sponge species ranged from 346 for *S. flabelliformis* to 379 MFs for *P. heteroaphis*.

Of the 503 MFs, 79 could be annotated using the RTL library with scores between 0.6 and 0.97. Another six MFs were annotated using the NIST 08 GC-MS library. A total of 85 aMFs were subjected to biological interpretation in the differential abundance analysis (see below in the “Shared or differential abundant molecular features” section and Additional file 6: Appendix file 1).

### Shared or differential abundant metabolites

**Fig. 1:**
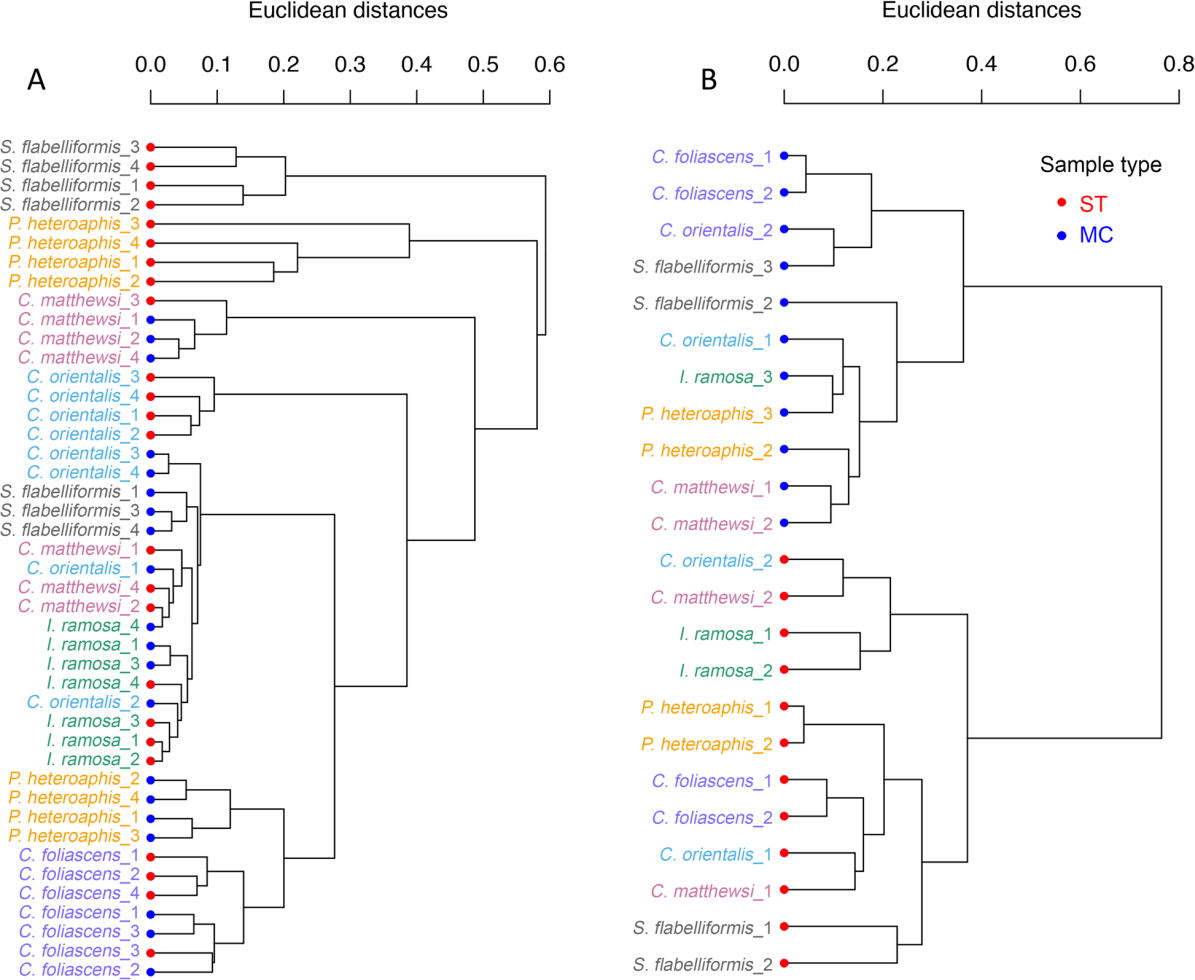
Cluster dendrograms of samples based on LC-MS/MS (**A**) or GC-MS (**B**) data. Symbol color indicates sample type. ST, whole sponge tissue; MC, microbial cell

The clustering dendrogram (Fig. 1) and PCA plot (Supplemental Fig. S1) of the LC-MS/MS data showed that metabolite profiles varied more between the factor “sponge species” than between “sample type”. This observation was supported by the PERMANOVA, which showed that sponge species have statistically significant differences in their metabolite profiles (*p* = 0.001, *R*^2^ = 0.370, *Df* = 5), while there was no statistical support for differences between sample types (*p* = 0.987) or the combination of the two factors (*p* = 0.999). Further comparison found statistical support for differences between all pairs of sponge species, except for *C. orientalis* versus *I. ramose* and *C. matthewsi* versus *I. ramose* (Supplemental Table S2).

Clustering dendrograms (Fig. 1) and PCA plot (Supplemental Fig. S1) of the GC-MS data showed that metabolite profiles varied more between sample types than across sponge species, which is different to the LC-MS/MS data. This was supported by a PERMANOVA, which showed that the factor “sample type” was the primary driver (*R*^2^ = 0.438, *p* = 0.001, *Df* = 1) and “sponge species” was the secondary contributor (*R*^2^ = 0.249, *p* = 0.001, *Df* = 5) to the statistical differences in metabolite profiles of samples.

**Fig. 2:**
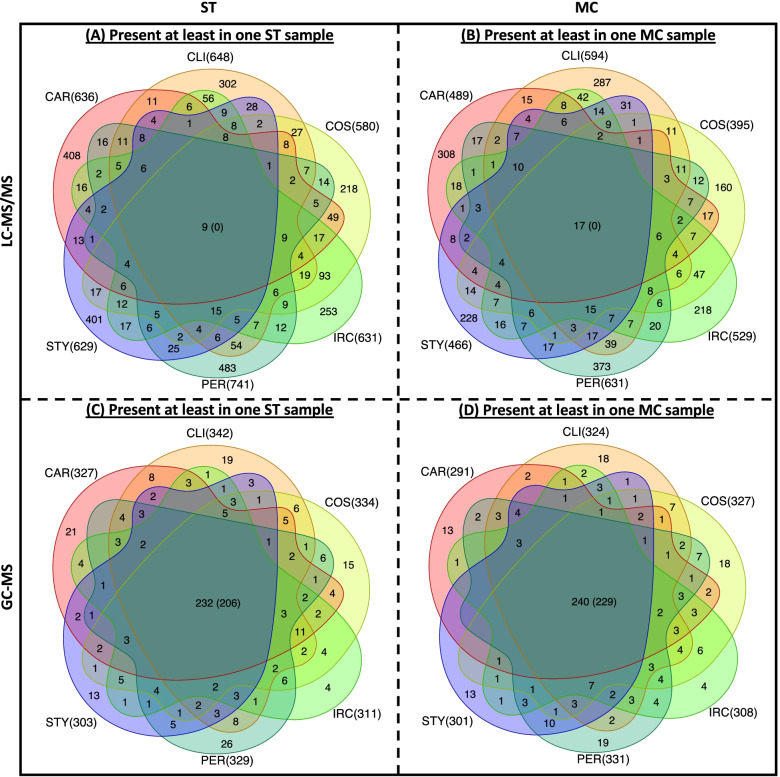
Venn diagrams of common and unique MFs across sponge species. The Venn diagram shows number of common and unique MFs of LC-MS/MS and GC-MS datasets that are present in at least one ST or MC sample of each species. The figure in brackets inside the overlapping area represents the number of MFs detected in every sample. The number of total MFs found in each species is shown in the brackets on the outside of the Venn diagrams. ST, whole sponge tissue; MC, microbial cell; CAR, *C. foliascens*; CLI, *C. orientalis*; COS, *C. matthewsi*; IRC, *I. ramosa*; PER, *P. heteroaphis*; STY, *S. flabelliformis*

Presence/absence analysis of the LC-MS/MS-based metabolite profiles showed that no or very few metabolites in either the ST or the MC samples were detected across all sponge species (Fig. 2). Specifically, 37‑65% of all MFs found in ST samples were unique to any given species. Only nine MFs were found in all sponge species, of which five could be annotated, and these include hymenialdisine, 2-methylbutyryl-carnitine, and eicosatrienoic acid methyl ester or related (stereo)-isomers (Table 2). Hymenialdisine is an alkaloid that was previously found in sponges and has been suggested to have antifouling activity [68]. 2-Methylbutyryl-carnitine, a short chain acylcarnitine, is produced by many eukaryotes, including sponges [69]. Eicosatrienoic acid methyl ester is an isomeric, methylated derivative of dihomo-γ-linolenic acid, which has been previously found in the sponge *Fasciospongia cavernosa* [70] and might be involved in host defense and immunity [71, 72].

**Table 2:** aMFs present in at least one ST or MC sample per sponge species. CAS RN refers to the registration number in the Chemical Abstract Service database, while match score refers to annotation confidence (cosine score between the experimental MS/MS spectra and the reference library MS/MS spectra). These spectral annotations correspond to level 2 based on MSI standards and could correspond to stereoisomers or closely related isomers

MF id	aMFs putatively annotated by GNPS workflow	CAS RN	Sample type	Match score
X21343	Didodecyl 3,3′-thiodipropionate oxide	17243140	ST	0.98
X2554	2-Methylbutyryl-carnitine	256928753	ST	0.73
X3329	Phe-Pro	N/A	ST	0.98
X6702	Hymenialdisine	N/A	ST	0.66
X8196	Eicosatrienoic acid methyl ester	21061109	ST	0.69
X1253	Val-Pro	N/A	MC	0.94
X20687	Didodecyl 3,3′-thiodipropionate (DLTDP)	123284	MC	0.99
X7396	Docosatetraenoic acid	28874580	MC	0.90

Between 38 and 63% of all LC-MS/MS-based MFs found in the MC samples were unique to any given species (Fig. 2). Seventeen MFs were found in all sponge species with three of them receiving an annotation (aMFs), including docosatetraenoic acid and didodecyl 3,3′-thiodipropionate (DLTDP) (Table 2)*.* The former is a compound with potential antibacterial, antibiofilm, and anti-inflammatory activities found in a sea anemone (*Stichodactyla haddoni*) [73] and sea hares (*Aplysia* sp.) [74], while the latter is an antioxidant [75] that has been isolated from the fungi *Geosmithia lavendulan* [76]. None of the remaining aMFs listed in Table 2 have previously been reported to occur in sponges or marine samples, nor do they have any known biological function.

The GC-MS-based metabolite profiles across sponge species showed that the majority of MFs were shared between the ST and MC samples and across sponge species (Fig. 2). Of the 206 and 229 MFs found across all ST and MC samples, respectively, 202 MFs were detected in all samples across both sample types, and only seven being unique to MC samples. None of these unique MFs could be assigned to known structures.

None of the metabolites found in current study matched those previously reported from the same sponge species in the MarinLit database [77], highlighting the high level of novel metabolic diversity found here. However, our general findings of distinct metabolite profiles across sponge species are consistent with patterns seen from the MarinLit database, which also showed only a limited or no overlap of metabolites between six sponge species (Supplemental Fig. S2 and Table S3).

Given that generally very distinct metabolite profiles were observed between sponge species in the LC-MS/MS dataset (Figs. 1 and 2), we next analyzed the differentially abundant metabolites (i.e., annotated differentially abundant MFs, aDAMs) between sample types (i.e., MC and ST) for each sponge species separately. About 62 ± 17% (range: 22‑85%) of MFs were normally distributed and homoscedastic (Supplemental Table S1) and therefore they were analyzed by unpaired t tests with the Benjamin-Hochberg (BH) corrected *p* value cut-off of < 0.05. The remaining MFs were analyzed by Mann-Whitney test with the same corrected *p* value cut-off. The number of DAMs ranged from eight for *I. ramosa* to 303 to *S. flabelliformis*, and the proportions of aDAMs ranged from 6% in *C. foliascens* to 25% in *I. ramosa* (Table 1). For most species, 84‑100% of the aDAMs had a higher relative abundance in the MC samples, except for *C. matthewsi*, where 88% of its aDAMs were relatively more abundant in the ST samples (Table 1 and Additional files 7 and 8: Appendix files 2 and 3).

**Fig. 3:**
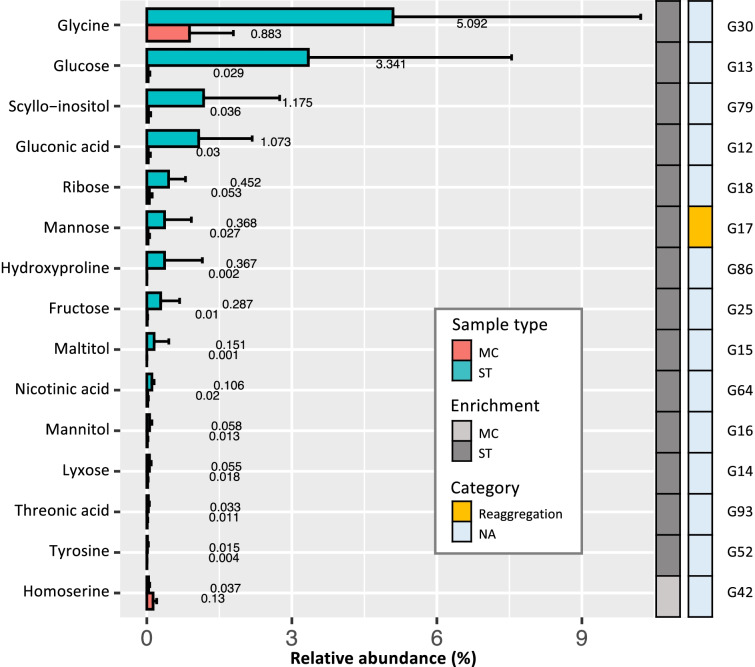
Histogram of aDAMs of the GC-MS analysis. The mean value of relative abundance was calculated based on the ion peak intensity of the molecule in each sample type. ST, whole sponge tissue; MC, microbial cell

Multivariate analysis on the GC-MS data showed that similar metabolite profiles were present across sponge species (Figs. 1 and 2) and therefore we analyzed DAMs between sample types across all sponge species. Due to the low proportion (about 10 ± 4%, range: 7‑13%) of MFs with normal distribution and homoscedasticity (Supplemental Table S1), all MFs were analyzed by the Mann-Whitney test and a BH corrected *p* value cut-off of < 0.05 was used. Sixty DAMs were found across all sponge species, with fifteen of them being annotated (aDAMs) (Fig. 3). A large proportion (93%) of these aDAMs was enriched in the ST samples (Additional file 6: Appendix file 1). All these aDAMs were next investigated for their potential biological or ecological role in the sponge holobionts.

### Compounds indicating metabolic interactions within the sponge holobionts

**Fig. 4:**
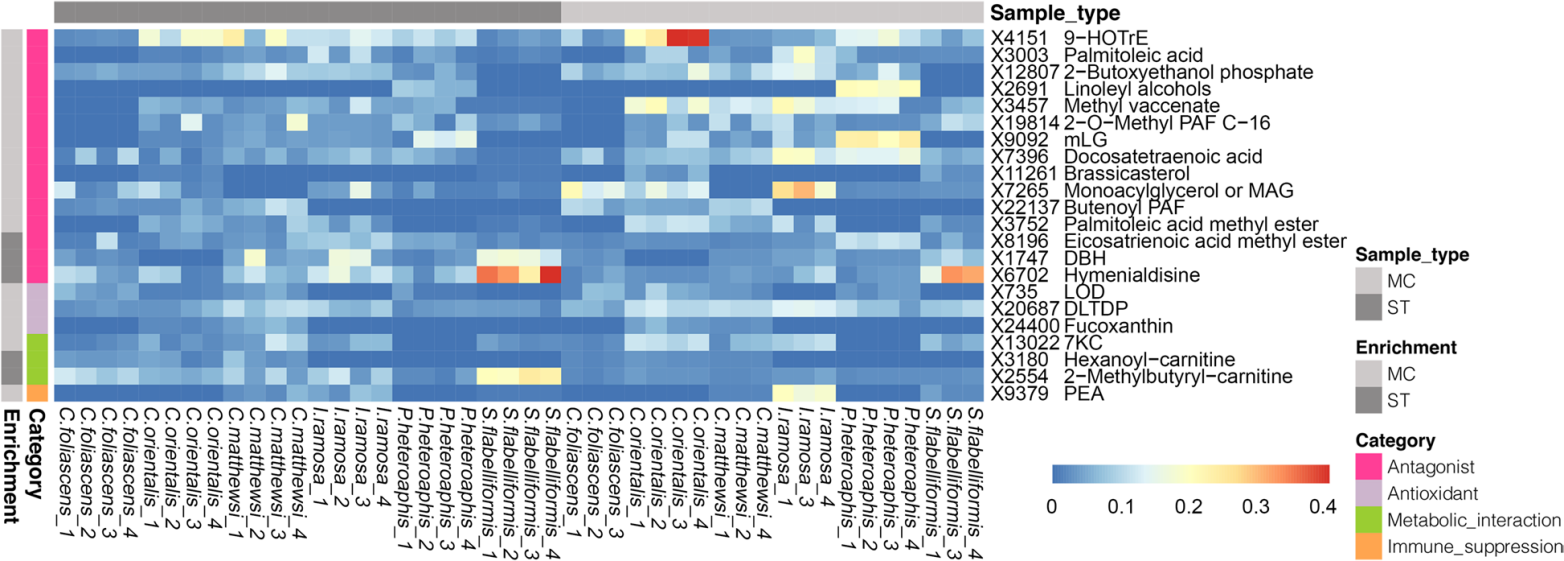
Heatmap of aDAMs of the LC-MS/MS analysis. Map color displays cube-root transformed, relative abundances. ST, whole sponge tissue; MC, microbial cell

In addition to the observation that 2-methylbutyryl-carnitine was broadly found in the ST samples across all sponge species (see above and Table 2), another short chain acylcarnitine, hexanoyl-carnitine (MF id: X3180), was found as an aDAM enriched in the ST samples of *C. foliascens* and *C. matthewsi* (Fig. 4). Carnitine (β-hydroxy-γ-N-trimethylammonium butyrate) can be used by some bacteria as a source for carbon, nitrogen, and energy, or as a protective molecule against fluctuations in salinity or temperature [78]. Carnitines therefore appear to be common metabolites in sponges that could be used by their associated microorganisms as a nutrient source. This is also supported by a recent metagenome-based observation that the sponge *Aplysina aerophoba* contains a group of symbiotic bacteria that are genetically adapted to metabolize carnitines [79].

Several carbohydrates including fructose, mannitol, gluconic acid, glucose, ribose, and scyllo-inositol (stereoisomer of inositol) (MF id: G25, G16, G12, G13, G18, and G79, respectively) were found to be enriched in the ST samples across all sponge species (Fig. 3). Genetic evidence has shown that sponge-associated gammaproteobacteria [80], *Anaerolineae* sp., *Caldilineae* sp. [81], and *Phyllobacteriaceae* [82] are capable of utilizing these carbohydrates, and sponges have been proposed to provide these as carbon and energy sources to their microbial symbionts [81]. This would be consistent with the enrichment and depletion of these carbohydrates in the sponge tissue and microbial cell fraction, respectively.

7-Ketocholesterol (7KC) (MF id: X13022) was found enriched in the MC samples of *C. orientalis*. Sterol biosynthesis is primarily associated with eukaryotes, and only rarely found in bacteria [83, 84]. 7KC is a major product of the reaction between cholesterol and oxygen radicals, and can cause cellular damage [85]. Interestingly, many bacteria can degrade 7KC by using it as sole nutrient [86, 87] and energy source [88]. 7KC has also been recently reported in the sponge *Axinella sinoxea* [89], and sterol-degrading microorganisms have been found in several marine sponges, such as *Sarcotragus* sp., *Petrosia* sp., and *Aplysina* sp. [90].

Together, these observations highlight the potential for specific metabolic interactions with benefits to the sponge-associated microorganisms. In the case of 7KC degradation, the interaction could be mutualistic, as it would remove a putative toxic compound from the host.

### Molecules with antioxidant activities

An enrichment of loliolide (LOD) (MF id: X735) and fucoxanthin (MF id: X24400) were found in the MC samples of *C. orientalis*. LOD is a monoterpenoid previously found in various plants, algae, and zebrafish, and has been described to possess antioxidant activities [91]. Fucoxanthin is a major non-provitamin A carotenoid with antioxidant activity [92], which is often found in brown seaweeds, diatoms, and golden algae [93], and has also recently been identified in a bacterial symbiont of the sponge *Callyspongia vaginalis* [94].

Generally, antioxidant compounds scavenge and degrade free radicals and other reactive oxygen species (ROS) that induce tissue damage [95], and sponges are considered to be one of the major sources of natural antioxidants [96]. A variety of antioxidants found in *C. orientalis* might have a role in protection against oxidative stress caused by zooxanthellae *Symbiodinium*, which has been reported to be abundant in and form symbiotic relationship with this sponge [97, 98]. The enrichment of these specific antioxidant compounds in the microbial cell fraction further support the notion that symbionts play a key role in providing protection against oxidative stress in sponges [11].

### Molecules with antagonist properties

Comparative analysis of the LC-MS/MS data showed that 1-*O*-hexadecyl-2-*O*-butenoyl-sn-glyceryl-3-phosphocholine (MF id: X22137) and 1-*O*-hexadecyl-2-*O*-methyl-sn-glyceryl-3-phosphorylcholine (MF id: X19814) were enriched in the MC samples of *C. foliascens* and *S. flabelliformis*, respectively. The former is known as a platelet activating factor (PAF), while the latter is a methyl-PAF with antibacterial activity also previously found in the sponge *A. sinoxea* [89]. PAFs are involved in various physiological processes, including antimicrobial defense [99, 100]. A recent study also found high levels of PAFs in the coral *Oculina patagonica* when exposed to *Vibrio* coral pathogens [101], indicating potential roles of PAFs in pathogen defense in both corals and sponges.

Several MFs annotated as PUFAs were found to be differentially abundant in either the whole sponge tissue or microbial cells. For example, 9-hydroxy-octadecatrienoic acid (9-HOTrE) (MF id: X4151) was found to be enriched in the MC samples of *C. orientalis*, while docosatetraenoic acid (MF id: X7396) was found to be enriched in the MC samples of *C. orientalis*, *P. heteroaphis*, and *S. flabelliformis*. In addition, eicosatrienoic acid methyl ester (MF id: X8196) (Table 2) was enriched in the ST samples of *C. matthewsi* (Fig. 4). The given annotation of these metabolites in untargeted mass spectrometry could also correspond to closely related (stereo)isomers. PUFAs however have been generally reported to play multiple critical roles in host defense, including antibacterial, antifungal, and antioxidant activities [72]. The antimicrobial activity provided by PUFAs could therefore be attributed to either the sponge tissues or their associated microbial cells, depending on the sponge species.

Both palmitoleic acid (MF id: X3003) and palmitoleic acid methyl ester (MF id: X3752) were found enriched in the MC samples of *C. orientalis* and *S. flabelliformis*. The former is a free fatty acid (FFA), while the latter is its fatty acid methyl ester (FAME). Again, the given specific annotation of long chain fatty acids could also reflect closely related (stereo)isomers. Bacterial symbionts of demosponges are known for their ability to synthesize short-chain C_15_-C_20_ monomethyl-substituted FFAs [42, 102], such as palmitoleic acid, which has been previously described in the sponges *Baikalospongia intermedia* [23] and *Axinella sinoxea* [24]. The C16:1δ6 isomer of palmitoleic acid is an effective inhibitor against gram-positive bacteria in human skin sebum [103]. Although specific functions of palmitoleic acid methyl ester are still not clear, they might act as an antimicrobial compound as increased antimicrobial activity has been found in some FAMEs in comparison to their non-methylated counterparts [104]. Another FAME, methyl vaccenate (MF id: X3457), was found to be enriched in the MC samples of *C. matthewsi* and has been described to have antimicrobial activity [25].

The MC samples of *C. orientalis* and *P. heteroaphis* were enriched with 1-monolinoleoyl-rac-glycerol (mLG) (MF id: X9092), which has antibacterial [105], antifungal [106], and antiviral activities [107]. 1-Palmitoyl-rac-glycerol (monoacylglycerol or MAG) (MF id: X7265) and linoleyl alcohols (MF id: X2691) were found to be enriched in the MC samples of *P. heteroaphis*. It has been reported that MAG has a strong inhibitory activity against biofilm formation [108], and linoleyl alcohols can effectively inhibit the growth of *Streptococcus mutans* [109]. An enrichment of brassicasterol (MF id: X11261) was found in the MC samples of *S. flabelliformis*. The phytosterol brassicasterol has an antiviral activity by inhibiting viral replication [110] and has previously been found in seaweed extracts [111]. 2-Butoxyethanol phosphate (MF id: X12807), a bioactive fatty acid with antagonistic activity [112, 113], was found to be enriched in the MC samples of *C. orientalis.*

Aside from the commonly found hymenialdisine (see above and Table 2), debromohymenialdisine (DBH) (MF id: X1747) was another compound enriched in whole sponge tissue of *C. matthewsi* and may be involved in preventing potential fouling organisms from settling or overgrowing the sponges. Both hymenialdisine and DBH have previously been isolated from spherulous cells of the sponge *Axinella* sp. [114] and have been found to exhibit antifouling activities against the mussel *Perna viridis* and the seaweed *Ulva prolifera* [68].

Many previous studies have not identified the true producers of antimicrobials within the sponge holobiont [30, 31]. In the current study, compounds with potential antagonistic properties appear to be commonly found in the microbial cell fractions and only occasionally in the whole sponge tissue. This suggests that these compounds are mainly produced by the microbial members in the sponge holobiont, and are potentially either involved in inter-microbial competitions or in defenses against intruding organisms.

### Molecules involved in cell-cell recognition and immune suppression in sponges

Comparative analysis of the GC-MS data showed that mannose (MF id: G17) was significantly more abundant in ST than in MC samples for all six sponge species (Fig. 3). Reaggregation is central to discrimination between self and non-self, a process that is vital for multicellular organisms and allows individuals to avoid invasion and parasitism from other organisms. Sponges can be dissociated to the cellular level and reaggregate afterwards, and an extracellular product named “aggregation factor (AF)” is responsible for this species-specific process [115]. Mannose was shown to be involved in the AF-mediated adhesion of the sponge *Microciona prolifera* and may therefore play an important role in the cell reaggregation process [116]. Enrichment of mannose in whole tissue of all species highlights its potentially universal role in cell-cell recognition and adhesion across sponges.

The MC samples of *I. ramosa* and *S. flabelliformis* were enriched with phenylethylamide (PEA) (MF id: X9379), a bacterial secondary metabolite also found in the bacterial genera *Xenorhabdus* and *Photorhabdus* and which causes immunosuppression against target insects by inhibiting eicosanoid biosynthesis [117]. It has also been isolated from the marine actinomycete *Salinispora arenicola* [118] and the soft coral *Sinularia flexibilis* [119], where its bioactivity was unconfirmed. The finding of PEA in sponge-associated microbial cells might indicate that their host species experience microbially induced immunosuppression.

## Conclusions

Comparative analysis of metabolomic data from whole tissue and microbial cell fractions of six marine sponge species revealed numerous metabolites potentially involved in biological activities that could contribute to the ecological function and survival of the holobionts. The putative annotations obtained for these metabolites allowed us to generate hypothesis on their function.

All sponge species investigated here appear to have metabolic interactions with their symbionts by providing them with 2-methylbutyryl-carnitine and carbohydrates, including scyllo-inositol, gluconic acid, ribose, fructose, and mannitol, as potential nutrients. The microbial symbionts appear to play a major role in response to oxidative stress as the antioxidant DLTDP was common to all symbiont communities, while LOD and fucoxanthin were specific to the symbiont communities of certain sponge species. Oxidative stress is common in shallow-water sponges as a result of photosynthesis-derived oxygen production by symbionts [8, 11, 120] and the associated microbial communities likely plays a role in preventing oxidative damage in the holobiont.

A number of putatively annotated metabolites are known to be involved in chemical defense such as eicosatrienoic acid methyl ester and hymenialdisine and those were common to the whole tissue of all sponge species. However, most chemicals with defensive properties appeared to originate from the microbial symbionts, including docosatetraenoic acid, which were found across all sponge species, or PAFs, mLG, and methyl vaccenate, which were restricted to specific sponge species. These molecules likely play a role in defending the sponge holobiont from fouling organisms [68] or microbial pathogens [73].

Finally, mannose was enriched in the tissue of all sponge species, which is notable as it likely plays a central role in recognizing self from non-self as part of the innate immune system. Microbial symbiosis might also induce immunosuppression as indicated by the high relative abundance of PEA in *I. ramosa* and *S. flabelliformis*. Sponges are increasingly recognized to have sophisticated immune-systems that could contribute to the discrimination of symbionts from food microorganisms [121, 122]. The stable relationship between sponge hosts and their associated symbionts might be established via the chemically mediated interactions observed here.

## Supplementary Information

**Additional file 1: Figure S1.** PCA plots for LC-MS/MS (A) and GC-MS (B) analysis. Symbol colour and shape indicate sponge species and sample type, respectively. ST: whole sponge tissue; MC: microbial cell.

**Additional file 2: Figure S2.** Number of common and unique sponge-produced natural metabolites from the MarinLit database for each sponge species. CAR: *C. foliascens*; CLI: *C. orientalis*; COS: *C. matthewsi*; IRC: *I. ramosa*; PER: *P. heteroaphis*; STY: *S. flabelliformis*.

**Additional file 3: Table S1.** Percentage of molecular features for which normality, homoscedasticity or both assumptions are met.

**Additional file 4: Table S2.** Pairwise PERMANOVA analysis based of Euclidian distances of normalized relative abundance of MFs of the LC-MS/MS dataset (including 24 ST and 20 MC samples). Bonferroni correction is used to correct for multiple test.

**Additional file 5: Table S3.** Sponge-derived natural products retrieved from the MarinLit database.

**Additional file 6: Appendix file 1.** Chemical annotation of MFs.

**Additional file 7: Appendix file 2.** Relative abundance of MFs and differential abundance analysis results.

**Additional file 8: Appendix file 3.** Differential abundance analysis of metabolites.

## Data Availability

The LC-MS/MS and GC-MS datasets generated during the current study are deposited in the MassIVE public repository under the accession number MSV000083475 and MSV000083743, respectively. The data that support the findings of this study are available in the supplementary materials.

## Abbreviations

LC-MS/MS: Liquid chromatography tandem mass spectrometry
GC-MS: Gas chromatography mass spectrometry
ST: Whole sponge tissue
MC: Microbial cell
MF: Molecular feature
aMF: Annotated molecular feature
DAM: Differentially abundant molecular feature
aDAM: Annotated differentially abundant molecular feature
PERMANOVA: Permutational multivariate analysis of variance
PCA: Principal component analysis
BH: Benjamin-Hochberg
PUFA: Polyunsaturated fatty acid
PAF: Platelet activating factor
FFA: Free fatty acid
FAME: Fatty acid methyl ester
