# Supplementary figures and images for: Comparative metabolomic analysis reveals shared and unique chemical interactions in sponge holobionts
Source: Microbiome. 2022 Feb 1;10:22. doi: 10.1186/s40168-021-01220-9 (PMC8805237; doi:10.1186/s40168-021-01220-9)

LC-MS/MS

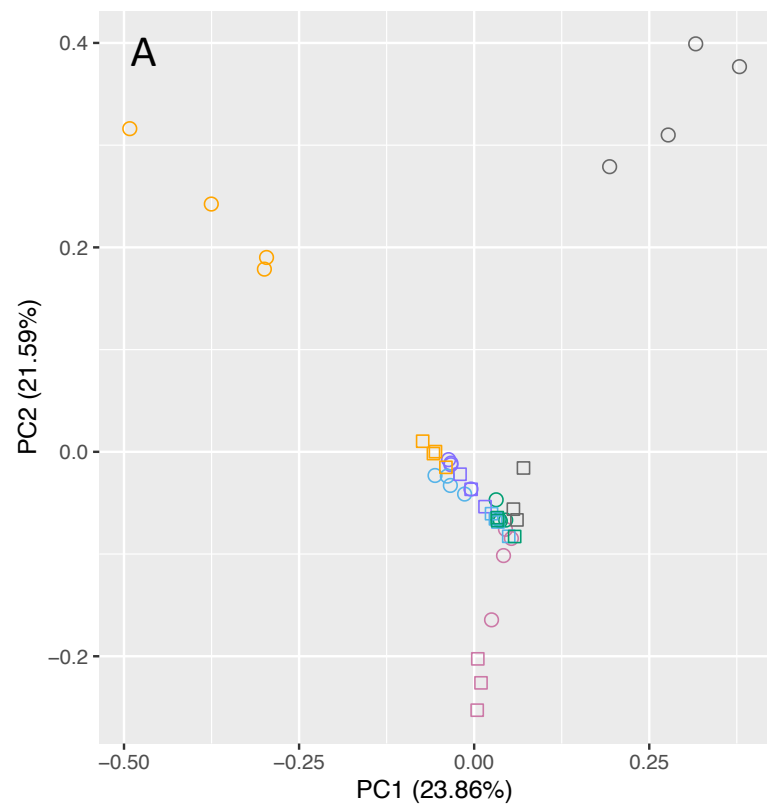

GC-MS

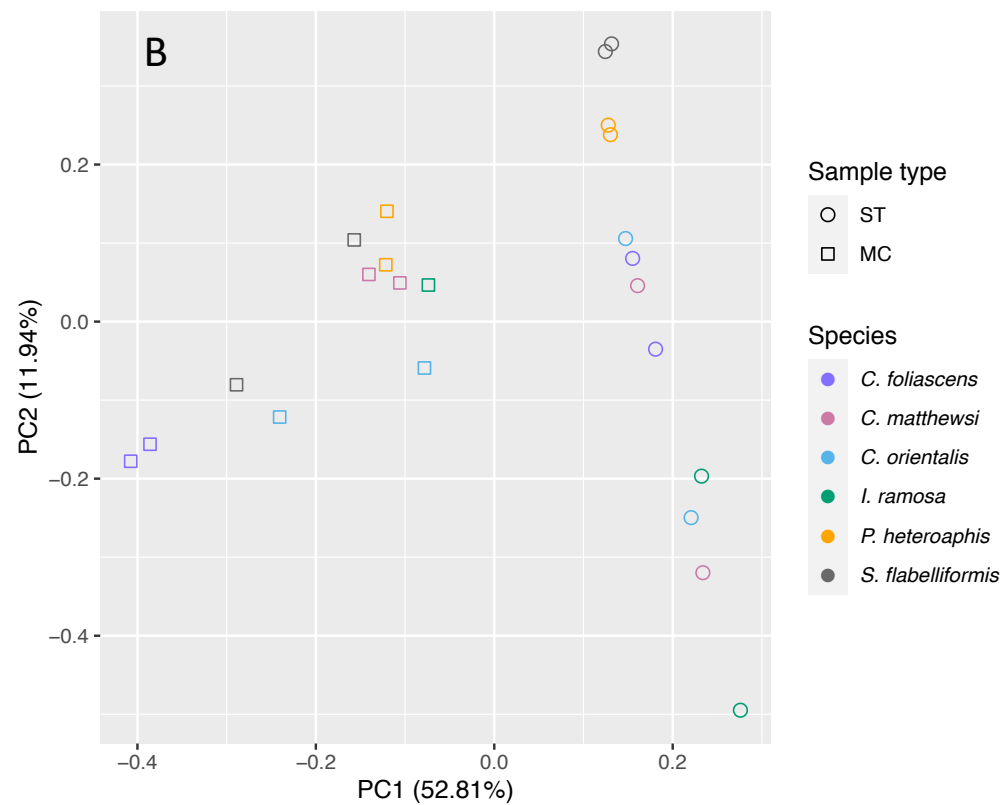

Supplement: Supplementary file 2 — Additional file 1: Figure S1. PCA plots for LC-MS/MS (A) and GC-MS (B) analysis. Symbol colour and shape indicate sponge species and sample type, respectively. ST: whole sponge tissue; MC: microbial cell. [file 40168_2021_1220_MOESM2_ESM.pdf]

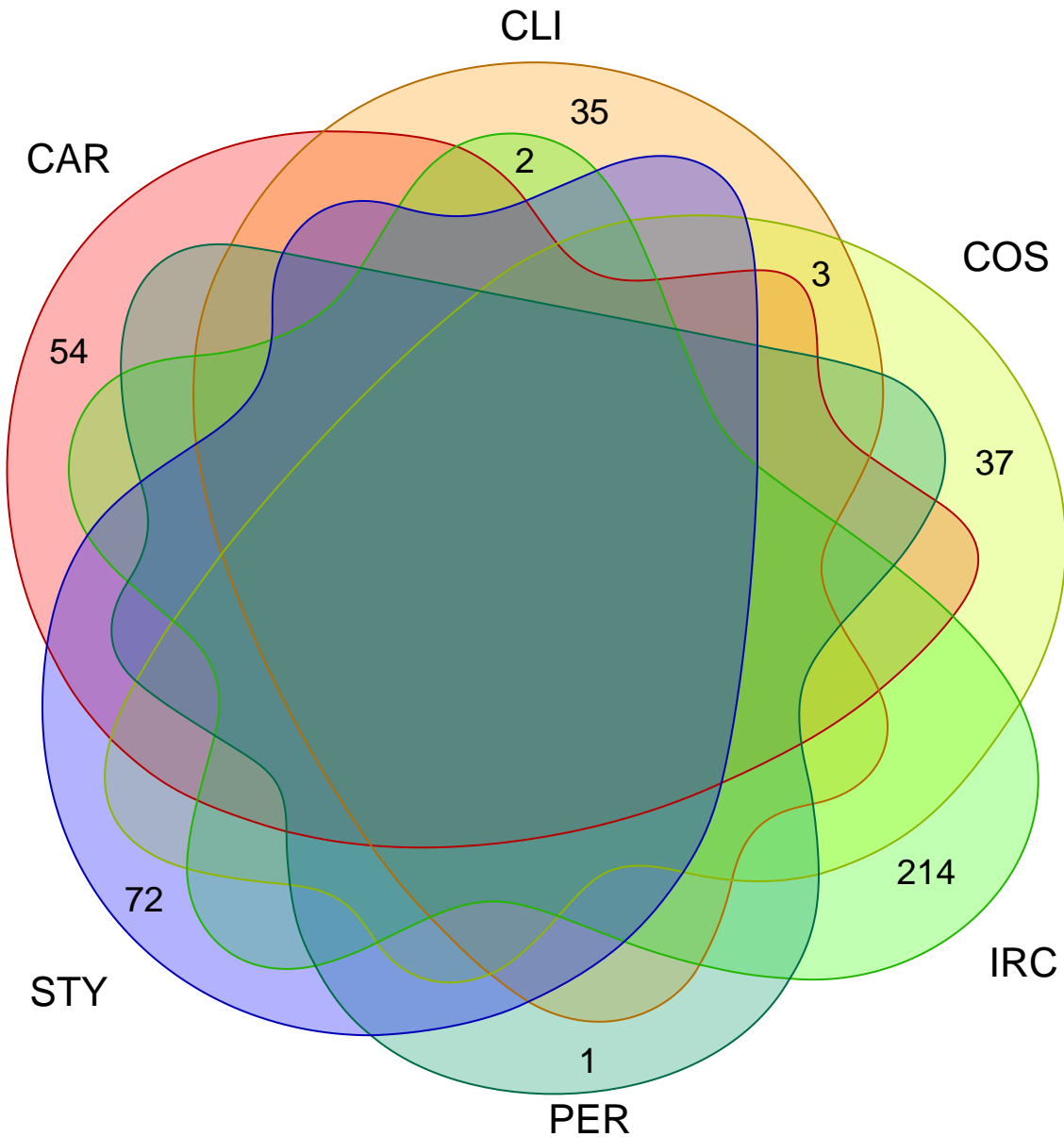

Supplement: Supplementary file 3 — Additional file 2: Figure S2. Number of common and unique sponge-produced natural metabolites from the MarinLit database for each sponge species. CAR: C. foliascens; CLI: C. orientalis; COS: C. matthewsi; IRC: I. ramosa; PER: P. heteroaphis; STY: S. flabelliformis. [file 40168_2021_1220_MOESM3_ESM.pdf]
